# Sarcopenia and Comorbidity Burden Independently Predict Cough Impairment in Hospitalized Patients: A Cross-Sectional Study

**DOI:** 10.3390/jcm15062332

**Published:** 2026-03-18

**Authors:** Marco Casciaro, Sara Manti, Delia Mammano, Antonella Gambadauro, Giorgio Basile, Chiara Tombetti, Silvano Cincotti, Francesco Nucera, Sebastiano Gangemi, Paolo Ruggeri

**Affiliations:** 1 Department of Clinical and Experimental Medicine, School and Operative Unit of Allergy and Clinical Immunology, University of Messina, 98125 Messina, Italy; marco.casciaro@unime.it (M.C.); sebastiano.gangemi@unime.it (S.G.); 2 Pediatric Unit, Department of Human Pathology in Adult and Developmental Age “Gaetano Barresi”, University of Messina, Via Consolare Valeria 1, 98125 Messina, Italy; gambadauroa92@gmail.com; 3 Pulmonology Unit, Department of Biomedical and Dental Sciences, Morphological and Functional Images (BIOMORF), University of Messina, 98125 Messina, Italy; delia.mammano@studenti.unime.it (D.M.); francesco.nucera@unime.it (F.N.); paolo.ruggeri@unime.it (P.R.); 4 Unit and School of Geriatrics, Department of Clinical and Experimental Medicine, University of Messina, 98125 Messina, Italy; giorgio.basile@unime.it; 5 Department of Mechanical, Energy, Management and Transport Engineering, University of Genoa, 16145 Genoa, Italy; chiara.trombetti@edu.unige.it (C.T.); silvano.cincotti@unige.it (S.C.)

**Keywords:** peak cough flow, sarcopenia, handgrip strength, respiratory muscle function, hospitalized patients, comorbidity, gender differences

## Abstract

**Background and Objectives:** Peak Cough Flow (PCF) is an objective measure of cough effectiveness, traditionally used in patients with neuromuscular disorders. Sarcopenia may also impair respiratory muscles, but its relationship with cough efficacy in hospitalized patients with respiratory diseases is not well established. This study investigated the correlation between PCF and sarcopenia indicators and evaluated the influence of comorbidities, anthropometric variables, and body position on PCF. **Methods:** A cross-sectional observational study was performed. PCF was measured using a portable peak flow meter in seated and supine positions. Sarcopenia was assessed through handgrip strength and validated questionnaires. Comorbidity burden was quantified using the Charlson Comorbidity Index (CCI). Nutritional status and sleep apnea risk were evaluated with the Mini Nutritional Assessment–Short Form (MNA-SF) and STOP-BANG questionnaire. Correlation analyses and linear regression were performed. **Results:** 53 patients were enrolled (mean age 72.6 ± 15.2 years; 64% male). Men showed significantly higher PCF values than women in both seated (*p* < 0.001) and supine positions (*p* < 0.001). Sarcopenic patients exhibited reduced PCF compared to non-sarcopenic subjects (*p* = 0.037). Handgrip strength was strongly correlated with PCF in seated and supine positions (*p* < 0.0001). CCI was negatively correlated with PCF (seated r^2^ = 0.17, *p* = 0.0021; supine r^2^ = 0.16, *p* = 0.0027). No significant associations were observed with BMI, MNA-SF, or STOP-BANG. Postural change resulted in comparable PCF reduction in men and women (ΔPCF: 20 ± 37.9 vs. 17 ± 37.9 L/min). **Conclusions:** Sarcopenia and comorbidity burden are significantly associated with reduced cough efficacy. Handgrip strength is a strong predictor of PCF, supporting routine PCF assessment beyond neuromuscular populations.

## 1. Introduction

Effective cough is a fundamental defensive mechanism that plays a crucial role in airway clearance and protection against respiratory infections [1]. Peak Cough Flow (PCF) measurement provides an objective assessment of cough efficacy and has been established as a valuable clinical parameter in respiratory care [2]. Despite its clinical significance, PCF evaluation has traditionally been limited to patients with neuromuscular disorders, in which cough impairment is a well-recognized risk factor for respiratory complications and mortality [3]. Recent evidence suggests that cough efficacy may be compromised in a broader range of hospitalized patients, particularly those with respiratory conditions [2]. However, systematic assessment of cough efficacy remains notably absent from routine clinical practice in non-neuromuscular patients admitted to pneumology units. This represents a significant gap in patient care, as impaired cough efficacy may contribute to prolonged hospitalization, increased risk of respiratory complications, and poorer clinical outcomes [4]. Sarcopenia, characterized by progressive loss of skeletal muscle mass and function, is increasingly recognized as a condition that extends beyond the musculoskeletal system to affect respiratory function [5]. The prevalence of sarcopenia in hospitalized patients, particularly among the elderly, is substantial and often underdiagnosed [6]. While the impact of sarcopenia on respiratory muscle strength and overall pulmonary function has been documented, the specific relationship between sarcopenia and cough efficacy remains poorly understood, especially in elderly patients with respiratory disorders [7]. Handgrip strength measurement has emerged as a practical and reliable surrogate marker for overall muscle strength and has been incorporated into sarcopenia assessment protocols [8]. Similarly, validated questionnaires provide valuable information on functional limitations and quality of life impairments associated with sarcopenia [9].

The integration of these assessment tools with PCF measurement offers a comprehensive approach to evaluating the potential relationship between muscle dysfunction and cough impairment. This study aims to investigate the correlation between cough efficacy, measured by PCF, and sarcopenia, assessed through standardized questionnaires and handgrip strength measurement, in patients hospitalized in a pneumology unit. By expanding PCF assessment beyond the traditional confines of neuromuscular disorders and exploring its relationship with sarcopenia, we aim to deepen understanding of cough dysfunction across a broader patient population and potentially identify new therapeutic targets, particularly in elderly patients with respiratory conditions.

## 2. Materials and Methods

An exploratory cross-sectional observational study was conducted to assess the impact of certain clinical and anthropometric variables on the efficacy of cough, measured by PCF, in hospitalized patients. It was analyzed how the length of stay in hospital, the presence of comorbidities and various indicators of sarcopenia could affect PCF measured in the sitting and supine positions. Furthermore, the difference between the two measurements in the sitting and supine positions of the PCF was considered to explore the possible effect of postural change on the ability to generate an effective cough. All patients of age ≥18 years admitted to the Pulmonology Unit, G. Martino Hospital, University of Messina, between January and March 2025 with a primary diagnosis of a respiratory diseases were considered eligible for participation. All patients admitted need to be able to understand and provide written informed consent (or availability of legal guardian), to maintain seated and supine positions for at least 5 min and to perform a voluntary cough maneuver on command. All patients admitted were in a hemodynamic stability (systolic blood pressure > 90 mmHg, heart rate 50–120 bpm, absence of unstable arrhythmias). Exclusion criteria were the presence of a severe cognitive impairment (Mini-Mental State Examination < 15 or inability to follow simple commands), undrained pneumothorax, recent thoracic or abdominal surgery (<4 weeks), recent rib or vertebral fractures (<4 weeks), severe uncontrolled pain preventing maneuver execution, tracheostomy, specific contraindications to increased intrathoracic pressure (e.g., untreated aortic aneurysm, recent cerebral hemorrhage), known neuromuscular disorders and conditions preventing handgrip measurement (e.g., severe rheumatoid arthritis of the hands, recent upper limb fractures).

The local Ethics Committee approved the study protocol (C.E.L. prot. n. 133-23 of 16 May 2024) and written informed consent was obtained from all participants or their legal guardians prior to enrollment.

The study sample consisted of consecutively admitted patients, all of whom underwent standardized clinical and functional assessments during their hospital stay.

Demographic and clinical data were collected from electronic medical records, including age, sex, body mass index (BMI), smoking history, and primary diagnosis.

The Mini Nutritional Assessment-Short Form (MNA-SF) and the STOP-BANG questionnaire were used to assess nutritional status and sleep apnea risk, respectively, categorized on a scale of 0 to 2 according to the level of risk or impairment.

Comorbidities were systematically assessed using the Charlson Comorbidity Index (CCI).

Duration of hospital stay was calculated from admission to discharge.

In-hospital mortality was recorded for all participants.

### 2.1. Cough Efficacy Assessment

PCF was measured using a portable peak flow meter (Mini-Wright Peak Flow Meter, Clement Clarke International Ltd., Wales, UK) connected to a face mask. Measurements were performed with patients in both seated and supine positions, following standardized procedures [10]. For each position, three consecutive measurements were taken with a one-minute rest period between attempts, and the highest value was recorded for analysis. PCF values were expressed in liters per minute (L/min). Participants were instructed to inhale maximally and then perform a forceful cough through the mask. Verbal encouragement was provided to ensure maximal effort. A PCF above 360 L/min is generally considered a normal, effective cough, while a PCF below 160 L/min indicates an inefficient cough, potentially leading to secretion retention and increased infection risk. A PCF between 160 and 270 L/min may indicate a risk of deterioration and increased vulnerability to respiratory complications [11].

### 2.2. Sarcopenia Assessment

Sarcopenia was evaluated using a multimodal approach combining muscle strength assessment. Handgrip strength was measured using a calibrated hydraulic hand dynamometer (Jamar Plus+, Performance Health, Downers Grove, IL, USA) according to the American Society of Hand Therapists protocol [12]. Measurements were taken with participants seated, shoulders adducted, elbow flexed at 90°, and forearm in neutral position. Three consecutive measurements were performed on each hand with a 60 s rest period between attempts, and the highest value was recorded for analysis. Participants were classified as sarcopenic based on handgrip strength cut-off values according to the European Working Group on Sarcopenia in Older People 2 (EWGSOP2) criteria12: <27 kg for men and <16 kg for women. Handgrip strength was additionally scored on a scale of 0 to 3 based on predefined thresholds: 0 = normal strength (men ≥ 27 kg, women ≥ 16 kg); 1 = mild reduction (men 20–26.9 kg, women 12–15.9 kg); 2 = moderate reduction (men 15–19.9 kg, women 8–11.9 kg); 3 = severe reduction (men < 15 kg, women < 8 kg).

### 2.3. Statistical Analysis

Sample size calculation was based on detecting a meaningful correlation between PCF and handgrip strength with 80% power and α level of 0.05. Continuous variables were presented as mean ± standard deviation or median (interquartile range) based on distribution normality. Categorical variables were expressed as absolute numbers and percentages. Correlations between PCF measurements (in both positions) and sarcopenia indices were assessed using Pearson’s or Spearman’s correlation coefficients, as appropriate. Simple linear regression models were conducted to evaluate the association between Peak Cough Flow (PCF) and individual predictors, including sex, age, and BMI. The differences in PCF values between specific patient subgroups (e.g., sarcopenic vs. non-sarcopenic) were analyzed using independent *t*-tests or Mann–Whitney U tests. A one-way analysis of variance (one-factor ANOVA) was performed to evaluate differences among non-sarcopenic individuals, sarcopenic men, and sarcopenic women. A *p*-value < 0.05 was considered statistically significant. All statistical analyses were performed using GraphPad Prism version 9.0 (GraphPad Software, LLC, Boston, MA, USA).

## 3. Results

Overall, demographic and clinical data of an enrolled population are reported in Table 1.

Respiratory diseases, their distribution within the sample and their heterogeneity detected in the enrolled population were reported in Table 2.

Comorbidities were reported in Table 3.

Data analysis revealed a substantial sex-related difference in PCF values. Globally, men exhibited higher mean PCF values than women in both the seated and supine positions. However, the postural change from seated to supine resulted in a comparable average reduction in PCF across both sexes. In the seated position, men reached a mean PCF of approximately 255 L/min (±109), whereas women averaged around 159 L/min (±53), a value that falls within the critical threshold (PCF < 160 L/min). In the supine position, mean values decreased in both groups, but the gap persisted: men maintained a mean PCF of about 235 L/min (±113), compared with 142 L/min (±56) in women. The ΔPCF was similar between groups, with an average reduction of approximately 20 L/min in men and 17 L/min in women. The regression analyses demonstrated that female sex and increasing age were significantly associated with lower PCF values in both seated and supine positions (Table 4).

These findings indicate that men exhibit stronger cough capacity, likely attributable to greater respiratory muscle mass.

Data analysis revealed no statistically significant correlation between BMI and PCF, either in the seated or supine position.

The correlation between BMI and PCF in the seated position was very weak and positive (Pearson’s r ≈ 0.112, *p* > 0.05). This indicates a slight increase in PCF with higher BMI, although the association is negligible. In the supine position, the correlation remained very weak and positive (r ≈ 0.158, *p* > 0.05), again suggesting a non-significant relationship. Considering the difference between seated and supine PCF (ΔPCF), the correlation with BMI was slightly negative (r ≈ −0.137, *p* > 0.05). This very weak, non-significant correlation suggested that higher BMI is associated with a minor reduction in PCF variation between positions, although this effect is likely due to chance.

Overall, although weak positive or negative trends were observed between BMI and PCF measures, none of these associations reached statistical significance. These findings suggested that BMI does not exert a meaningful influence on cough strength or on its variation between seated and supine positions.

### 3.1. MNS-SF Score and PCF

The analysis of the association between Mini Nutritional Assessment-Short Form (MNA-SF) scores and PCF provided insights into the potential association between nutritional status and PCF.

In the seated position, a weak positive correlation was observed (r ≈ 0.26), with a *p*-value close to the threshold of significance (*p* ≈ 0.056). This suggested that better nutritional status may favour more effective cough strength, although statistical evidence was not sufficient to confirm this association. In the supine position, the correlation remained weak (r ≈ 0.24) and was not statistically significant (*p* ≈ 0.08), confirming a trend but not strong enough to draw firm conclusions. Regarding the variation between positions (ΔPCF), the correlation with MNA-SF was virtually absent (r ≈ 0.045) and not significant (*p* ≈ 0.75).

Overall, the findings suggested that nutritional status may influence cough strength in both seated and supine positions. However, none of the observed correlations reached statistical significance. Further data or a larger sample size would be required to clarify this potential association.

### 3.2. STOP-BANG Score and PCF

Comparing STOP-BANG scores with PCF, it emerged that in the seated position, the correlation was virtually absent (r ≈ −0.01) and not significant (*p* ≈ 0.93). In the supine position, a similar result was observed, with no correlation (r ≈ −0.03) and a non-significant *p*-value (*p* ≈ 0.81). For postural variation (ΔPCF), the correlation was negligible (r ≈ 0.06) and not significant (*p* ≈ 0.67).

No correlation was found between STOP-BANG scores and cough strength, either in the seated or supine position, or in the variation between the two. Thus, the risk of obstructive sleep apnea did not appear to affect the ability to generate an effective cough.

### 3.3. Comorbidities and PCF

The association between patient comorbidities, assessed via the CCI, and PCF values in seated and supine positions was evaluated. The potential impact of the respiratory diseases within the sample was also investigated.

**Figure 1:**
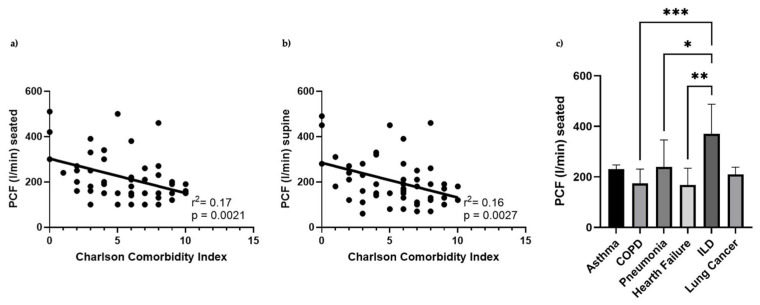
(**a**,**b**) Correlation between Charlson Comorbidity Index (CCI) and Peak Cough Flow (PCF) in seated (left panel) and supine (right panel) positions. Each dot represents an individual patient. The trend lines demonstrate that higher comorbidity burden is significantly associated with reduced cough efficacy in both positions. (**c**) Correlation between Peak Cough Flow (PCF) and comorbidity distribution: asthma, Chronic Obstructive Pulmonary Disease; pneumonia, hearth failure, Interstitial Lung Disease, and cancer. * *p* < 0.05, ** *p* < 0.01, *** *p* < 0.001.

In the seated position, a significant negative correlation was observed (r^2^ = 0.17; *p* = 0.0021), indicating that higher comorbidity burden is associated with reduced cough strength (Figure 1). This suggests that an increased CCI negatively impacts patients’ ability to expectorate, representing a clinically relevant risk factor in the management of complex patients. Although the correlation is moderate in magnitude, the *p*-value confirmed its statistical significance. In the supine position, the negative correlation remained statistically significant (r^2^ = 0.16; *p* = 0.0027), showing that higher comorbidity burden is similarly associated with decreased cough strength in the supine posture (Figure 1).

Moreover, the relationship between distribution of comorbidities and PCF showed a significant association for COPD, pneumonia and heart failure (*p* < 0.05).

Reduced cough effectiveness in patients with high comorbidity, particularly in the supine position, might contribute to an increased risk of respiratory complications. These findings highlighted the importance of comprehensive, multidimensional assessment in patients with multiple comorbidities.

### 3.4. Sarcopenia and PCF

The assessment of the association between sarcopenia and PCF showed that sarcopenic individuals had lower mean PCF values compared with non-sarcopenic subjects, both in seated and supine positions.

Data analysis revealed that mean PCF values differed significantly among the three groups. Non-sarcopenic subjects exhibited a mean PCF of 242.8 L/min when seated and 226.4 L/min when supine. Sarcopenic men showed mean values of 223.8 L/min (seated) and 204.8 L/min (supine), while sarcopenic women had markedly lower means of 131.4 L/min (seated) and 104.3 L/min (supine), well below the critical threshold of 160 L/min. This pattern indicates a clear trend of reduced PCF in the presence of sarcopenia, particularly among women.

**Figure 2:**
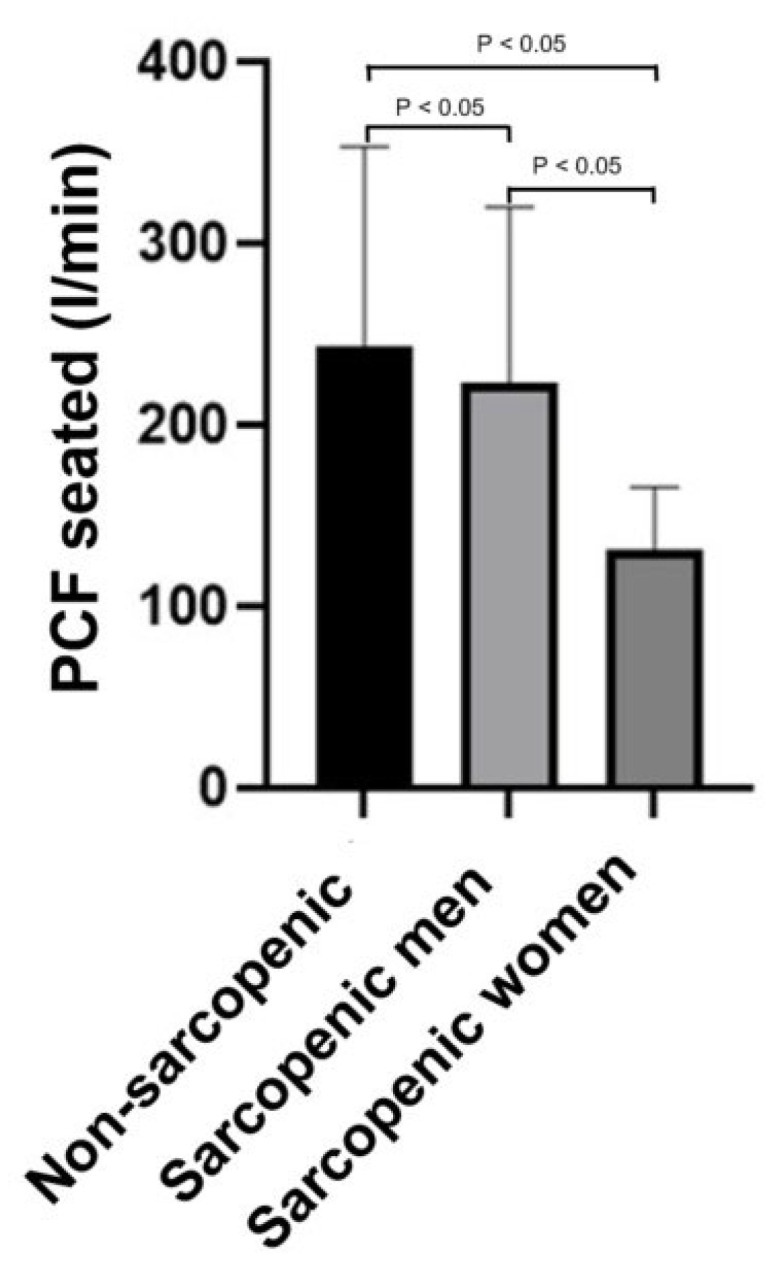
Comparison of Peak Cough Flow (PCF) in seated position among three groups: non-sarcopenic individuals, sarcopenic men, and sarcopenic women. Data are presented as mean ± standard deviation.

One-way ANOVA yielded F = 3.53 for seated measurements and F = 4.09 for supine measurements, both exceeding the critical value (F crit = 3.18), with corresponding *p*-values of 0.0367 and 0.0226, confirming that the differences were statistically significant (Figure 2). Post hoc Tukey HSD test demonstrated that the significant difference was primarily driven by the comparison between non-sarcopenic individuals and sarcopenic women (*p* < 0.05).

These findings supported the hypothesis that sarcopenia is associated with a significant reduction in PCF, with a more pronounced impact observed in sarcopenic women. This evidence underscored the importance of early recognition of sarcopenia and sex-specific assessment in clinical and functional evaluations.

### 3.5. Handgrip and PCF

Analysis of the correlation between handgrip strength and PCF values revealed a significant association between muscle strength and peak cough flow, both in the seated and supine positions.

**Figure 3:**
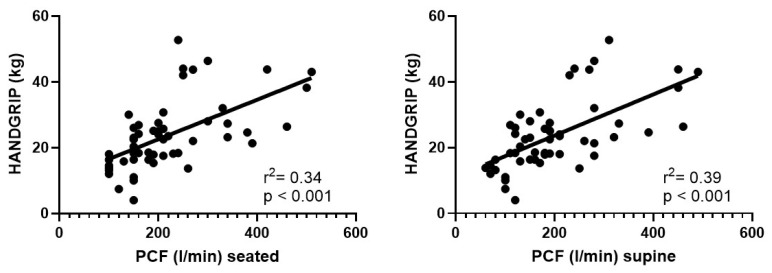
Correlation between handgrip strength and Peak Cough Flow (PCF) in seated (**left panel**) and supine (**right panel**) positions. Each dot represents an individual patient. The trend lines demonstrate that greater peripheral muscle strength is significantly associated with higher cough efficacy in both positions, supporting the link between peripheral and respiratory muscle function.

In the seated position, a moderately strong positive correlation was observed (r^2^ = 0.34), which was highly significant (*p* < 0.0001) (Figure 3). This indicates that lower handgrip strength is associated with reduced cough strength, suggesting that diminished overall muscle strength is reflected in reduced cough effectiveness. In the supine position, the correlation was even stronger, with a coefficient of r ≈ 0.63, indicating a strong positive association: individuals with lower muscle strength exhibited markedly reduced cough strength (Figure 3). This correlation was also highly significant (*p* ≈ 0.0000005), reinforcing the notion of a direct link between global and respiratory muscle strength. By contrast, when considering the difference between seated and supine PCF (ΔPCF), the correlation with muscle strength was very weak and negative (r ≈ −0.16) and not statistically significant (*p* ≈ 0.26). This suggests that muscle strength is not clearly related to changes in PCF between positions.

The positive association between muscle strength and PCF, observed in both seated and supine positions, were strong and highly statistically significant. These findings indicated that individuals with greater muscle strength are better able to generate an effective cough, resulting in higher PCF values in both positions, thereby confirming the link between peripheral and respiratory muscle strength. However, muscle strength did not appear to substantially influence the degree of PCF variation across positions. This suggested that the reduction in PCF in the supine position is more likely attributable to biomechanical and respiratory factors rather than peripheral muscle strength alone.

## 4. Discussion

Our cross-sectional observational study provides novel insights into the association between cough efficacy, as measured by PCF, and various indicators of sarcopenia in hospitalized patients. To our knowledge, this is one of the first studies to specifically investigate this association, extending the evaluation of cough effectiveness beyond the traditional context of neuromuscular disorders to a broader population of patients with respiratory conditions.

Our findings reveal the role of anthropometric parameters, handgrip strength and comorbidity burden on cough efficacy.

Gender-specific differences in the impact of comorbidities on cough efficacy were striking in our cohort. Biological sex also independently influenced PCF, with men demonstrating higher values, consistent with previous studies showing greater respiratory muscle mass and lung volumes in males [13,14]. This sex-specific effect may be attributed to differences in muscle mass distribution, hormonal influences, and baseline respiratory capacity. Women with cardiac comorbidities, particularly acute myocardial infarction and congestive heart failure, exhibited dramatic reductions in cough performance [15], while men demonstrated greater resilience to these conditions. This gender disparity has been noted in broader research on respiratory outcomes but has not previously been documented specifically for cough efficacy [16]. Yan Zhang et al. reported similar gender differences in respiratory muscle function among heart failure patients but did not assess cough parameters [15].

The lack of a significant association between PCF and BMI suggests that body mass alone does not strongly determine cough strength, possibly because BMI does not differentiate between lean and fat mass, which have contrasting effects on respiratory function. Similarly, nutritional status assessed by MNA-SF showed weak or nonsignificant correlations with PCF [17]. While there was a trend suggesting better nutritional status might support cough strength, the evidence was insufficient to confirm a statistically significant effect, possibly due to sample size limitations. The lack of significant associations between nutritional screening tools and PCF was unexpected, given the established association between nutritional status and respiratory muscle function. This might reflect the complexity of the association between nutritional parameters and specific respiratory functions, like cough, or limitations in the sensitivity of the screening tools used. Standard nutritional assessment tools were poor predictors of respiratory muscle function in patients with chronic respiratory diseases [18]. Our finding that cerebrovascular disease significantly impacted cough efficacy, particularly in women in the supine position (−86% in the first functional transition), adds to the growing body of evidence linking neurological impairment to respiratory dysfunction. Studies by Park et al. (2018) have documented similar associations in stroke patients but focused primarily on swallowing function rather than cough effectiveness [19].

The observed negative correlation between the CCI and PCF highlights the clinical relevance of multimorbidity in reducing respiratory effectiveness. Patients with higher comorbidity burden may have compromised lung function, reduced mobility, or chronic inflammation, all of which could contribute to a weaker cough. The cumulative burden of comorbidities emerged as a powerful predictor of cough impairment, with patients having three or more comorbidities showing significant deterioration across the entire functional trajectory. This finding is consistent with the concept of multimorbidity as a determinant of functional decline, as documented by Wijnant et al. (2023) in their study of frailty in elderly patients with respiratory conditions [20]. However, our study extends this observation specifically to cough function, an aspect not previously investigated in multimorbidity research.

Notably, postural changes from sitting to the supine position revealed important functional vulnerabilities that may have clinical implications. The more pronounced negative effects of comorbidities and sarcopenia indicators in the supine position suggest that this position unmasks functional limitations that might compensate for in the sitting position. The reduction in cough efficiency in the supine position is likely driven by biomechanical factors, such as diaphragmatic displacement and reduced lung volumes, rather than peripheral muscle function alone. Similar postural effects have been documented by Gloeckl et al. (2023) for general respiratory parameters but not specifically for cough efficacy [21].

The lack of association between length of hospital stay and cough efficacy suggested that this parameter alone may not be a useful predictor of functional respiratory outcomes. This differs from findings by Verduri et al. (2025), who reported correlations between hospital length of stay and various respiratory parameters, although they did not specifically assess cough function [22].

Peripheral muscle strength and sarcopenia emerged as the strongest and most significant predictors of PCF. This supports the well-established notion that overall muscular function is closely linked to respiratory performance, likely because both peripheral and respiratory muscles share structural and functional characteristics. Individuals with reduced muscle strength, particularly sarcopenic patients, exhibited markedly lower PCF values.

Furukawa et al. (2025) similarly found that quality of respiratory muscle function, rather than mass, was more predictive of clinical outcomes in Chronic Obstructive Pulmonary Disease (COPD) patients, although they did not specifically assess cough efficacy [23].

The observed inverse relationship between handgrip strength and PCF improvement probabilities represents another counterintuitive finding. Patients with lower handgrip strength had a higher probability of improved cough function, particularly during the first functional transition (+31% in the sitting position). This contrasts with studies by Lee et al. (2020) [24] and Bahat et al. (2021) [25], who reported positive correlations between handgrip strength and various respiratory parameters in elderly populations. However, these studies did not specifically examine cough effectiveness. Our findings might reflect compensatory mechanisms or adaptations in patients with reduced peripheral muscle strength, who may develop alternative strategies to generate effective cough pressures.

### Clinical Implications

Our findings demonstrate that 78% of hospitalized pneumology patients exhibit suboptimal cough efficacy (PCF < 270 L/min), with 40% falling below the critical 160 L/min threshold. We recommend routine PCF screening at admission using portable peak flow meters to identify high-risk patients, particularly sarcopenic women and those with high comorbidity burden, more sophisticated assessments of muscle quality, such as ultrasound-derived echogenicity or specific biomarkers of muscle dysfunction, might provide further insights into the mechanisms underlying the observed associations.

## 5. Conclusions

Our study emphasizes the multifactorial nature of cough strength, integrating musculoskeletal, sex-specific, and comorbidity-related factors. An early identification and intervention for sarcopenia could improve cough efficiency and potentially reduce respiratory complications, particularly in women. Moreover, interventions aiming to enhance overall muscle strength, such as resistance training, may have a dual benefit for both peripheral and respiratory muscle function, reducing patient frailty [26]. Despite very sparse evidence, nutritional optimization could play a supportive, albeit secondary, role in maintaining cough capacity, especially in older or frail patients [27,28,29]. Finally, management strategies for patients with high comorbidity should include assessing and supporting cough effectiveness, particularly in supine or bedridden positions, to prevent pulmonary complications such as atelectasis or infections.

## Figures and Tables

**Table 1:** Demographic and clinical data of an enrolled population.

Variables	Mean ± SD
Age (years)	72.60 ± 15.23
BMI (kg/m^2^)	27.11 ± 7.25
Days of Hospitalization	14.67 ± 12.65
Pack-Years	34.04 ± 34.95
CCI	5.40 ± 2.80
HR (bpm)	85.26 ± 14.97
SBP (mmHg)	130.60 ± 20.11
Respiratory Rate (breaths/min)	19.55 ± 3.16
Seated PCF (L/min)	220.60 ± 102.90
Supine PCF (L/min)	201.70 ± 105.80
ΔPCF (L/min)	18.87 ± 37.91
Arm Circumference (cm)	28.60 ± 6.13
Calf Circumference (cm)	33.49 ± 4.92
Neck Circumference (cm)	40.58 ± 4.70
Handgrip Strength (kg)	23.78 ± 10.58
**MNA-SF**	**9.89 ± 2.87**
**STOP-BANG Score**	**3.94 ± 1.43**
	N (%)
SEX	
Male	34 (64)
Female	19 (36)
Smoking exposure	
Never Smoker	11 (21)
Current Smoker	23 (43)
Former Smoker	19 (36)
Nutritional status	
Normal	18 (34)
At Risk of Malnutrition	25 (47)
Malnourished	10 (19)
Peak Cough Flow	
Optimal > 360 L/min	6 (11)
Intermediate 270–360 L/min	6 (11)
Low 160–270 L/min	20 (38)
Critical < 160 L/min	21 (40)
Sarcopenia	
Sarcopenic	25 (47)
Non-Sarcopenic	28 (53)
Risk of Obstructive Sleep Apnea Syndrome	
Low Risk	5 (10)
Intermediate Risk	33 (62)
High Risk	15 (28)

BMI: Body Mass Index; CCI: Charlson Comorbidity Index; HR: Heart Rate; SBP: Systolic Blood Pressure; PCF: Peak Cough Flow; MNA-SF: Mini Nutritional Assessment-Short Form.

**Table 2:** List of respiratory diseases detected in the enrolled population.

Diseases	Nr. Patients
• Pneumonia	14
• AECOPD	13
• Bronchiectasis	2
• OSA	1
• Lung Cancer	4
• ILD	5
• Hearth Failure	9
• Obesity Hypoventilation Syndrome	4
• Pulmonary Hypertension	1
• Thoracic Trauma	1
**Respiratory failure**	
• Acute Respiratory Failure	35
• Acute on Chronic Respiratory Failure	15
• No Respiratory Failure	3

AECOPD: Acute Exacerbation of Chronic Obstructive Pulmonary Disease; OSA: Obstructive Sleep Apnea; ILD: Interstitial Lung Disease.

**Table 3:** List of comorbidities detected in the enrolled population.

Comorbidities	N	%
Acute Myocardial Infarction	5	9.43
Congestive Heart Failure	16	30.19
Peripheral Vascular Disease	6	11.32
Cerebrovascular Disease	6	11.32
Dementia	0	0.0
Chronic Pulmonary Disease	26	49.06
Connective Tissue Disease	0	0.0
Peptic Ulcer	0	0.0
Liver Disease	2	3.77
Diabetes	23	43.4
Hemiplegia	0	0.0
Moderate/Severe Renal Disease	14	26.42
Solid Tumor with Metastasis	2	3.77
Leukemia	0	0.0
Lymphoma	0	0.0
AIDS	0	0.0
Arterial Hypertension	42	79.25

AIDS: Acquired Immune Deficiency Syndrome.

**Table 4:** Linear Regression Analyses for Peak Cough Flow (PCF).

Predictor	Variable	β	SE	t	*p*-Value	R^2^
**Sex (1 = female)**	Seated PCF	−95.23	26.62	−3.58	<0.001	0.201
	Supine PCF	−92.89	27.69	−3.35	0.002	0.181
**Age (years)**	Seated PCF	−2.60	0.87	−2.97	0.005	0.148
	Supine PCF	−2.98	0.88	−3.39	0.001	0.184
**BMI (Kg/m^2^)**	Seated PCF	1.60	1.97	0.81	0.423	0.013
	Supine PCF	2.31	2.02	1.15	0.257	0.025

BMI = body mass index; PCF = peak cough flow; β = unstandardized regression coefficient; SE = standard error; t = t-value; R^2^ = R squared.

## Data Availability

The original data presented in the study are openly available in the text.

## Abbreviations

The following abbreviations are used in this manuscript:

BMI	Body mass index
CCI	Charlson Comorbidity Index
COPD	Chronic Obstructive Pulmonary Disease
EWGSOP2	European Working Group on Sarcopenia in Older People 2
MIP	Maximal inspiratory pressure
MEP	Maximal expiratory pressure
MNA-SF	Mini Nutritional Assessment–Short Form
PCF	Peak Cough Flow

## References

[1] Chang A.B. (2006). The physiology of cough. Paediatr. Respir. Rev..

[2] Brennan M., McDonnell M.J., Duignan N., Gargoum F., Rutherford R.M. (2022). The use of cough peak flow in the assessment of respiratory function in clinical practice—A narrative literature review. Respir. Med..

[3] Farrero E., Antón A., Egea C.J., Almaraz M.J., Masa J.F., Utrabo I., Calle M., Verea H., Servera E., Jara L. (2013). Guidelines for the Management of Respiratory Complications in Patients with Neuromuscular Disease. Arch. Bronconeumol. Engl. Ed..

[4] Stoev K., Wirth R., Labeit B., Muhle P., Suntrup-Krueger S., Dziewas R., Trampisch U.S., Lueg G., Pourhassan M. (2025). Prognostic value of cough force measured by peak expiratory flow in a 4-year longitudinal cohort study of geriatric patients with oropharyngeal dysphagia. Front. Aging.

[5] Sato S., Miyazaki S., Tamaki A., Yoshimura Y., Arai H., Fujiwara D., Katsura H., Kawagoshi A., Kozu R., Maeda K. (2023). Respiratory sarcopenia: A position paper by four professional organizations. Geriatr. Gerontol. Int..

[6] Bertschi D., Kiss C.M., Beerli N., Kressig R.W. (2021). Sarcopenia in hospitalized geriatric patients: Insights into prevalence and associated parameters using new EWGSOP2 guidelines. Eur. J. Clin. Nutr..

[7] Bone A.E., Hepgul N., Kon S., Maddocks M. (2017). Sarcopenia and frailty in chronic respiratory disease: Lessons from gerontology. Chron. Respir. Dis..

[8] Bahat G., Kilic C., Altinkaynak M., Akif Karan M. (2020). Comparison of standard *versus* population-specific handgrip strength cut-off points in the detection of probable sarcopenia after launch of EWGSOP2. Aging Male.

[9] Geerinck A., Beaudart C., Reginster J.Y., Locquet M., Monseur C., Gillain S., Bruyère O. (2021). Development and validation of a short version of the Sarcopenia Quality of Life questionnaire: The SF-SarQoL. Qual. Life Res..

[10] Kulnik S.T., MacBean V., Birring S.S., Moxham J., Rafferty G.F., Kalra L. (2015). Accuracy of portable devices in measuring peak cough flow. Physiol. Meas..

[11] Bach J.R., Saporito L.R. (1996). Criteria for extubation and tracheostomy tube removal for patients with ventilatory failure: A different approach to weaning. Chest.

[12] Sousa-Santos A.R., Amaral T.F. (2017). Differences in handgrip strength protocols to identify sarcopenia and frailty—A systematic review. BMC Geriatr..

[13] Cruz-Jentoft A.J., Bahat G., Bauer J., Boirie Y., Bruyère O., Cederholm T., Cooper C., Landi F., Rolland Y., Sayer A.A. (2019). Sarcopenia: Revised European consensus on definition and diagnosis. Age Ageing.

[14] Dominelli P.B., Molgat-Seon Y. (2022). Sex, gender and the pulmonary physiology of exercise. Eur. Respir. Rev..

[15] Zhang Y., Lin Z., Chen Y., Hong L., Shen X. (2024). Factors related to pre-operative cough strength in cardiac surgical patients: A cross-sectional study. Heart Lung.

[16] Han M.K., Postma D., Mannino D.M., Giardino N.D., Buist S., Curtis J.L., Martinez F.J. (2007). Gender and Chronic Obstructive Pulmonary Disease: Why It Matters. Am. J. Respir. Crit. Care Med..

[17] Mikiya R., Momoki C., Habu D. (2019). Factors associated with diminished cough intensity in community-dwelling elderly using day care services: A pilot study. J. Aging Res. Clin Pract..

[18] Kaluźniak-Szymanowska A., Krzymińska-Siemaszko R., Deskur-Śmielecka E., Lewandowicz M., Kaczmarek B., Wieczorowska-Tobis K. (2021). Malnutrition, Sarcopenia, and Malnutrition-Sarcopenia Syndrome in Older Adults with COPD. Nutrients.

[19] Park M.K., Lee S.J. (2018). Changes in Swallowing and Cough Functions Among Stroke Patients Before and After Tracheostomy Decannulation. Dysphagia.

[20] Wijnant S.R.A., Benz E., Luik A.I., Rivadeneira F., Voortman T., Brusselle G.G., Lahousse L. (2023). Frailty Transitions in Older Persons with Lung Function Impairment: A Population-Based Study. J. Gerontol. Ser. A.

[21] Gloeckl R., Zwick R.H., Fürlinger U., Jarosch I., Schneeberger T., Leitl D., Koczulla A.R., Vonbank K., Alexiou C., Spruit M.A. (2023). Prescribing and adjusting exercise training in chronic respiratory diseases—Expert-based practical recommendations. Pulmonology.

[22] Verduri A., Roberto T., Pierluigi D., Jonathan H., Giovanni G., Jovana M., Valentina R., Cristina M., Enrico C., Bianca B. (2025). Respiratory Muscle Dysfunction and Associated Risk Factors Following COVID-19-Related Hospitalisation. Life.

[23] Furukawa Y., Miyamoto A., Asai K., Tsutsumi M., Hirai K., Ueda T., Toyokura E., Nishimura M., Sato K., Yamada K. (2025). Respiratory Muscle Strength as a Predictor of Exacerbations in Patients with Chronic Obstructive Pulmonary Disease. Respirology.

[24] Lee S.E., Park J.-H., Kim K.-A., Kang Y.-S., Choi H.S. (2020). Association Between Sarcopenic Obesity and Pulmonary Function in Korean Elderly: Results from the Korean National Health and Nutrition Examination Survey. Calcif. Tissue Int..

[25] Bahat G., Altinkaynak M., Karan M.A. (2021). Handgrip strength cut-offs to define sarcopenia in Turkish population. Aging Clin. Exp. Res..

[26] Kongsgaard M., Backer V., Jørgensen K., Kjær M., Beyer N. (2004). Heavy resistance training increases muscle size, strength and physical function in elderly male COPD-patients—A pilot study. Respir. Med..

[27] Collins P.F., Yang I.A., Chang Y.-C., Vaughan A. (2019). Nutritional support in chronic obstructive pulmonary disease (COPD): An evidence update. J. Thorac. Dis..

[28] Henrot P., Dupin I., Schilfarth P., Esteves P., Blervaque L., Zysman M., Fares G., Maurice H., Pascal P., Patrick B. (2023). Main Pathogenic Mechanisms and Recent Advances in COPD Peripheral Skeletal Muscle Wasting. Int. J. Mol. Sci..

[29] Maniscalco M., Fuschillo S., Ambroino P., Candia C., di Domenico A., Lombardi C., Motta A., Ambrosino N. (2026). Skeletal muscle dysfunction in chronic obstructive pulmonary disease: MiRNAs, myokines and exercise. ERJ Open Res..

